# An ecological association of number of civil servants and physicians with prefectural-level rapid COVID-19 vaccination of older people in Japan

**DOI:** 10.1265/ehpm.22-00145

**Published:** 2023-04-07

**Authors:** Kimiko Tomioka, Midori Shima, Keigo Saeki

**Affiliations:** Nara Prefectural Health Research Center, Nara Medical University, Kashihara, Nara, Japan

**Keywords:** COVID-19, Vaccination, Older people, Civil servants, Physicians, Ecological studies, Japan

## Abstract

**Background:**

Civil servants and physicians play an important role in combating COVID-19. However, it is unclear whether the number of civil servants and physicians is associated with rapid COVID-19 vaccine uptake among older people (i.e., smoother rollout of priority vaccination for older people).

**Methods:**

Using Poisson regression models of the generalized estimating equations, we examined the ecological association of the number of civil servants and physicians with prefectural-level rapid COVID-19 vaccination in older people. Prefectural-level data were based on publicly available government surveys. The outcome variable was the proportion of fully vaccinated people aged 65 and older on the day with the largest standard deviation across 47 prefectures (i.e., July 6, 2021). The explanatory variable was the number of civil servants and physicians per population by prefecture.

**Results:**

After adjusting for population density, influenza vaccination coverage, socioeconomic factors, natural environmental factors, health indicators, and the number of civil servants and physicians, in all 3 models, prefectures with the highest number of civil servants and physicians had faster COVID-19 vaccine uptake than prefectures with the lowest number. A significant trend between higher staffing levels and more rapid vaccination was observed for the number of physicians in all 3 models, but for the number of civil servants only in one model.

**Conclusion:**

We found that COVID-19 vaccine uptake among older people was more rapid in prefectures with more civil servants and physicians per population, with the number of physicians having a stronger association. This study may point the way to future areas of research on vaccine policies that include other age groups and infectious diseases.

**Supplementary information:**

The online version contains supplementary material available at https://doi.org/10.1265/ehpm.22-00145.

## Background

Vaccinations are the most important and effective means of preventing infectious diseases [1, 2]. As the spread of the coronavirus disease 2019 (COVID-19) continues, it is an urgent task to promote vaccination as a national policy and expand vaccination coverage among the population, giving priority to vaccination of high-risk groups such as older people [2, 3].

It has been pointed out that the effectiveness of COVID-19 pandemic prevention and management is highly dependent on the effort and capacity of civil servants [4, 5]. Specifically, civil servants have a crucial part to play in pandemic countermeasures, such as collecting information, deploying personnel, and providing information on the prevention and control of COVID-19 to citizens [5]. Additionally, in Japan, the COVID-19 pandemic is a disaster response. Therefore, the implementation of initial vaccination, which is an important means of coping with the COVID-19 pandemic, was not only handled by the department in charge of peacetime vaccination operations, but also supported by other departments. As a result, local governments were able to secure the necessary personnel for COVID-19 vaccination [6]. Therefore, the number of civil servants per population may have been related to the initial rapid rollout of vaccinations. Moreover, a previous Japanese study reported that older people who have a family physician were about twice as likely to receive pneumococcal and influenza vaccines as those who do not [7]. Because previous influenza vaccination was associated with a higher rate of COVID-19 vaccine acceptance [8], the number of physicians per population may affect COVID-19 vaccine uptake among older people.

In Japan, regarding the priority of the initial vaccination for COVID-19, the government prioritized healthcare professionals, followed by older people aged 65 and older, and then those with pre-existing conditions and workers in nursing homes, based on the level of risk of severe disease and the securing of the health-care system [6, 9]. Vaccinations for healthcare professionals began in mid-February 2021, followed by vaccinations for older people in April [10, 11]. People under the age of 64 were vaccinated when supplies of vaccine became available in each prefecture. Regarding the vaccination framework, the government is responsible for securing and distributing vaccines and providing scientific information on vaccines to the public. Prefectures are responsible for cooperating in securing and setting up vaccination systems so that municipalities can smoothly administer vaccinations. Municipalities are responsible for implementing vaccination programs, encouraging residents to get vaccinated, and setting up mass vaccination centers [6, 9, 12].

Given the importance accorded to the role of civil servants and physicians, we decided to conduct an ecological study to examine the cross-sectional association of the number of civil servants and physicians per population with rapid COVID-19 vaccination in older people (i.e., smoother rollout of priority vaccination for older people).

## Methods

### Study design

This study is a prefectural-level ecological study using data published by the Government. The Government has introduced the “Vaccination Record System” (commonly known as VRS), a centralized database that provides real-time information on the population’s COVID-19 vaccination status. The Digital Agency has made this information publicly available as open data. We analyzed publicly available vaccination coverage of COVID-19 based on VRS as an outcome.

### Rapid COVID-19 vaccination

Since April 12, 2021, the Digital Agency has published daily the total number of vaccinations for all age groups and the breakdown of those aged 65 and older for the first and second vaccinations by prefecture [13]. Since the data released by the Digital Agency is in newline delimited JavaScript Object Notation format, the research team at Sapporo Medical University School of Medicine has processed it into an easy-to-use Excel format, and released it as open data on vaccination coverage by prefecture since May 3, 2021 [14, 15]. In this study, we used data on the vaccination status of people aged 65 and older published by the research team. The cumulative number of persons aged 65 and older who received a second dose of vaccine was then divided by the population aged 65 and older. In calculating the proportion of fully vaccinated people, we used the 2021 estimated population [16].

**Fig. 1:**
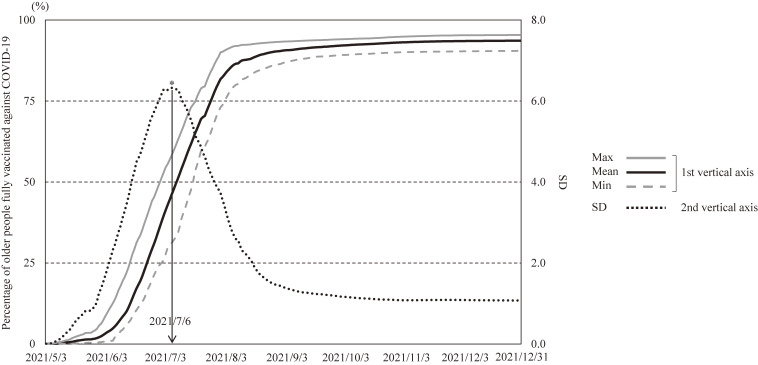
Daily percentage of older people aged 65 and older fully vaccinated against COVID-19 from May 3, 2021 to December 31, 2021. SD: standard deviation of vaccination coverage across 47 prefectures. *: Time point with the largest standard deviation.

We chose the time period when regional dispersion of the proportion of fully vaccinated older people was greatest, which is estimated to represent regional differences in rapid vaccination. That is, the standard deviation (SD) of the proportion of fully vaccinated older people in 47 prefectures was calculated on a daily basis, and identified the day when the SD was largest. The day with the largest SD was July 6, 2021 (daily progression of the proportion of fully vaccinated older people is shown in Fig. 1).

Therefore, in this study, the outcome variable was defined as the proportion of fully vaccinated people aged 65 and older on the day with the largest standard deviation across 47 prefectures (i.e., July 6, 2021).

### Number of civil servants and physicians

The Survey of Local Government Officials’ Salaries has been conducted by the Ministry of Internal Affairs and Communications with the aim of obtaining basic data on the salary system for civil servants [17]. This survey covers not only the salaries of local government officials, but also the number of employees. In this study, we used the number of local public employees by prefecture based on the 2020 Survey of Local Government Officials’ Salaries as the number of civil servants in each prefecture. The number of local public employees by prefecture in 2020 is further classified into the number of employees by organization. Therefore, using this data, we conducted an additional analysis stratifying civil servants into prefectural civil servants and municipal civil servants. Moreover, using the census population data as of October 1, 2020 [18], we calculated the number of civil servants by prefecture per 1,000 people. In this study, the number of civil servants per 1,000 people was defined as the number of civil servants.

The Statistics of Physicians, Dentists, and Pharmacists produced biennially by the Ministry of Health, Labour and Welfare surveys the number of physicians engaged in medical facilities [19]. The latest published data is from 2020. The 2020 Statistics of Physicians, Dentists, and Pharmacists used the 2020 census population data (as of October 1, 2020) released by the Statistics Bureau of Japan [18] when calculating the number of physicians per population. We adopted the data on the number of physicians per 100,000 population by prefecture, based on the 2020 Statistics of Physicians, Dentists, and Pharmacists [19]. In this study, the number of physicians per 10,000 people was defined as the number of physicians.

Regarding medical resources other than the number of physicians, we adopted the number of clinics, hospitals, and nurses per 100,000 population, referring to previous studies [20, 21]. For the number of clinics and hospitals, we used data from the 2020 Survey of Medical Institutions [22]. For the number of nurses, we used data from the 2020 Report on Public Health Administration and Services [23]. The number of hospitals per 100,000 population does not include the number of psychiatric hospitals and tuberculosis hospitals.

### Covariates

With reference to previous studies [2, 3, 21, 24–26], we selected the following three categories as important confounding factors in the association of the number of civil servants and physicians with rapid vaccination: prefectural-level socioeconomic factors, natural environmental factors, and health indicators. Socioeconomic factors included household income, educational status, and Gini coefficient. Natural environmental factors included annual mean temperature, annual mean relative humidity, and annual sunshine hours. Health indicators included all-cause mortality, cancer incidence rate, and per capita medical costs. Because this study was an ecological study using data from 47 prefectures, a maximum of 5 covariates could be entered [27]. As a way to solve the situation where the number of covariates that can be entered into one model is limited and more covariates must be used, three models were set up and the variables to be entered into each model were devised as follows:

1. Population density is an important covariate variable for comparison by prefecture [21, 28], and rapid COVID-19 vaccination may be associated with general vaccination coverage during normal times [8]. That is, population density and influenza vaccination coverage are essential confounders and should be included in each model.
2. Because there is a correlation between variables in each category (e.g., the Pearson’s correlation coefficient between annual mean relative humidity and annual sunshine hours was minus 0.62), each model should contain one variable from one category.
3. After checking the correlation between variables, the Pearson’s correlation coefficient between per capita medical costs and household income was minus 0.67, indicating that it would be better not to put them into the same model.

Based on the above, in all models, population density and influenza vaccination coverage were used as covariates. Then, for each model, we chose one socioeconomic factor, one natural environmental factor, and one health indicator as covariates. In Model 1, in addition to population density and influenza vaccination coverage, we added household income, annual mean temperature, and all-cause mortality to covariates. In Model 2, we added educational status, annual mean relative humidity, and cancer incidence rate to covariates. In Model 3, we added the Gini coefficient, annual sunshine hours, and per capita medical costs to covariates. These variables were obtained from publicly available databases. The details of definition and data source of covariates are shown in Additional file 1. The reasons why each variable was adopted as a covariate in this study are presented in Additional file 2.

### Statistical analysis

We used the multivariable Poisson regression model of the generalized estimating equations to estimate the association of the number of civil servants and physicians with rapid COVID-19 vaccination by prefecture. The independent variable was the number of civil servants and the number of physicians in each prefecture. The 47 prefectures were classified into quintile groups according to their number of civil servants, and the 1st quintile group with the lowest number of civil servants was defined as the reference group. The number of physicians was also divided into quintile groups in the same way as the number of civil servants, and the 1st quintile group with the fewest number of physicians was used as the reference group. We set the logarithm of the cumulative number of people aged 65 and older who received two doses of the COVID-19 vaccination as of July 6, 2021 by prefecture as the outcome variable, using the offset term to account for (the log to base e) the population by prefecture. We calculated a prevalence ratio (PR) and a 95% confidence interval (CI) for rapid COVID-19 vaccination (i.e., the proportion of fully vaccinated older people, as of July 6, 2021).

First, we calculated a crude PR for rapid COVID-19 vaccination. Next, in the adjusted Model 0, we adjusted for the 5 covariates (logarithm of population density, influenza vaccination coverage, household income, annual mean temperature, and all-cause mortality). Then, in the adjusted Model 1, we adjusted for the number of civil servants and physicians, along with the 5 covariates previously mentioned. That is, we mutually adjusted for the number of civil servants and physicians, in addition to the variables in Model 0. In the adjusted Model 2, we adjusted for logarithm of population density, influenza vaccination coverage, educational status, annual mean relative humidity, cancer incidence rate, and the number of civil servants and physicians. In the adjusted Model 3, we adjusted for logarithm of population density, influenza vaccination coverage, Gini coefficient, annual sunshine hours, per capita medical costs, and the number of civil servants and physicians. There were no variables with a variance inflation factor exceeding 5.0, and the three adjusted models had no problem of multicollinearity.

In additional analyses, we used the number of other health resources (i.e., the number of clinics, the number of hospitals, or the number of nurses) as a substitute for the number of physicians to examine the association of the number of health resources per 100,000 population with rapid COVID-19 vaccination in older people. Moreover, in additional stratified analyses, we divided the number of civil servants into prefectural civil servants and municipal civil servants and examined the association with rapid vaccination in older people.

We performed the analyses by age group. Statistical analyses were performed using the IBM SPSS Statistics Ver. 27 for Windows (Armonk, New York, US), and a significant level was set at 0.05 (two-tailed test).

## Results

The proportion of fully vaccinated people aged 65 and older was 44.6%, as of July 6, 2021. In Japan, the total number of civil servants in 2020 was 2,764,094 (the number of civil servants per 1,000 population was 21.9), and the total number of physicians engaged in medical facilities in 2020 was 323,700 (the number of physicians per 10,000 population was 25.7). The proportion of fully vaccinated people and the number of civil servants and physicians by prefecture are presented in Additional file 3.

Prefectures with a higher number of civil servants tended to have a lower population density, a higher influenza vaccination coverage, a lower household income, a lower level of educational status, a higher annual mean relative humidity, and a higher all-cause mortality, and a higher level of per capita medical costs. Prefectures with a higher number of physicians tended to have a lower household income, a higher Gini-coefficient, a higher annual mean temperature, a higher cancer incidence rate, a higher level of per capita medical costs, and a higher proportion of older people who received two doses of the COVID-19 vaccine (Table 1).

**Table 1:** Median of each variable by quintile groups of number of civil servants and physicians

	**Number of civil servants per 1,000 population**						**Number of physicians per 10,000 population**					
	
	**Q1**	**Q2**	**Q3**	**Q4**	**Q5**		**Q1**	**Q2**	**Q3**	**Q4**	**Q5**	
**Cut-off values for quintile groups**	**16.7–21.0**	**21.1–24.2**	**24.4–26.1**	**26.2–29.5**	**29.7–36.2**		**17.8–22.3**	**22.4–24.6**	**25.0–26.7**	**27.7–29.7**	**30.8–33.8**	
	Med (IQR)	Med (IQR)	Med (IQR)	Med (IQR)	Med (IQR)	*P* ^a^	Med (IQR)	Med (IQR)	Med (IQR)	Med (IQR)	Med (IQR)	*P* ^a^
Logarithm of population density	7.28 (1.31)	5.83 (0.43)	5.39 (0.44)	5.33 (0.83)	4.85 (0.55)	<0.001	6.15 (2.22)	5.71 (0.74)	5.39 (0.60)	5.53 (0.71)	5.58 (1.17)	0.963
Influenza vaccination coverage (%)	50.0 (8.6)	53.5 (3.9)	57.8 (2.4)	57.3 (2.6)	56.4 (4.2)	<0.001	53.6 (7.5)	54.9 (1.6)	56.6 (5.8)	57.4 (3.4)	53.6 (5.2)	0.593
Household income (100,000 yen)	45.0 (0.5)	43.9 (3.1)	41.4 (4.6)	40.2 (5.2)	41.8 (5.6)	0.009	44.7 (3.4)	45.0 (2.8)	41.8 (5.2)	41.0 (5.6)	39.0 (4.9)	0.004
Educational status^b^ (%)	58.4 (7.0)	56.3 (8.3)	47.9 (3.3)	46.9 (10.4)	46.6 (7.2)	<0.001	51.3 (9.3)	51.5 (7.0)	55.3 (12.3)	51.0 (11.3)	52.5 (2.3)	0.721
Gini coefficient	28.6 (2.9)	28.0 (1.2)	27.5 (1.4)	28.0 (1.9)	28.5 (3.0)	0.895	27.0 (1.3)	28.3 (1.7)	27.6 (2.6)	27.7 (1.9)	28.5 (2.2)	0.040
Annual mean temperature (°C)	17.0 (0.8)	16.8 (1.3)	16.1 (2.7)	17.4 (2.3)	15.8 (4.5)	0.464	15.0 (2.9)	15.4 (3.9)	16.1 (2.0)	17.4 (1.3)	17.5 (1.2)	<0.001
Annual mean relative humidity (%)	69.0 (3.0)	69.0 (5.0)	72.0 (5.0)	72.0 (6.0)	76.0 (6.0)	0.003	72.0 (6.0)	73.5 (9.0)	75.0 (9.0)	71.0 (3.0)	71.0 (2.0)	0.380
Annual sunshine hours (100 hours)	20.4 (1.4)	21.6 (2.9)	19.5 (3.9)	20.7 (4.1)	17.3 (5.8)	0.241	18.8 (4.5)	19.6 (3.8)	20.1 (4.5)	21.1 (2.8)	20.4 (2.9)	0.109
All-cause mortality (1,000 persons)	1.68 (0.13)	1.61 (0.06)	1.64 (0.14)	1.72 (0.11)	1.74 (0.08)	0.023	1.68 (0.18)	1.66 (0.09)	1.67 (0.08)	1.65 (0.15)	1.70 (0.12)	0.409
Cancer incidence rate (1 million)	37.7 (2.3)	39.1 (2.8)	37.9 (1.7)	40.1 (2.4)	38.7 (3.2)	0.241	38.2 (1.3)	37.4 (1.3)	37.9 (3.5)	39.9 (1.9)	39.9 (1.4)	0.012
Per capita medical costs (10,000 yen)	32.0 (1.0)	35.6 (4.7)	34.5 (6.9)	39.9 (3.5)	36.9 (4.2)	<0.001	32.6 (2.7)	33.5 (2.1)	37.6 (2.6)	39.9 (3.1)	39.5 (5.8)	<0.001
COVID-19 vaccination status^c^ (%)	39.3 (8.0)	44.5 (11.3)	48.0 (3.2)	45.2 (5.3)	50.1 (7.6)	0.067	38.8 (4.7)	47.5 (11.3)	44.1 (10.0)	46.8 (5.6)	50.1 (5.9)	<0.001

IQR, interquartile range; Med, median; Q1, 1st quintile group; Q2, 2nd quintile group; Q3, 3rd quintile group; Q4, 4th quintile group; Q5, 5th quintile group.

^a^*P*-value based on the Jonckheere-Terpstra trend test.

^b^Proportion of upper secondary graduates going to further education.

^c^Proportion of fully vaccinated people aged 65 and older, as of July 6, 2021.

In all modes, there was a consistent pattern: Higher PRs were found in prefectures with the highest number of civil servants and physicians (Table 2). For the number of civil servants, the adjusted PR (95% CI) of the 5th quintile group was 1.21 (1.04–1.40) in Model 1, 1.23 (1.07–1.42) in Model 2, and 1.25 (1.09–1.42) in Model 3, compared to the 1st quintile group. For the number of physicians, the adjusted PR (95% CI) of the 5th quintile group was 1.24 (1.12–1.38) in Model 1, 1.21 (1.14–1.29) in Model 2, and 1.21 (1.10–1.33) in Model 3, compared to the 1st quintile group. Regarding *P* for trend, the number of physicians had significant results in all models: *P* for trend was <0.001 in all models. In contrast, the number of civil servants showed a significant result only in Model 1: *P* for trend was 0.042 in Model 1, 0.177 in Model 2, and 0.080 in Model 3.

**Table 2:** Association of number of civil servants and physicians with rapid COVID-19 vaccination in older people

**Explanatory** **variables**	**Crude model**	**Adjusted model 0**	**Adjusted model 1**	**Adjusted model 2**	**Adjusted model 3**
	**PR (95% CI)**	**PR^a^ (95% CI)**	**PR^b^ (95% CI)**	**PR^c^ (95% CI)**	**PR^d^ (95% CI)**
Number of civil servants per 1,000 population					
Q1	1.00	1.00	1.00	1.00	1.00
Q2	1.05 (0.93–1.18)	1.10 (0.98–1.22)	1.06 (0.95–1.20)	1.08 (0.95–1.24)	1.09 (0.97–1.22)
Q3	1.14 (1.03–1.25)	1.19 (1.06–1.35)*	1.20 (1.06–1.35)*	1.22 (1.08–1.37)*	1.23 (1.09–1.38)*
Q4	1.00 (0.88–1.14)	1.15 (1.02–1.30)*	1.07 (0.92–1.25)	1.09 (0.94–1.27)	1.10 (0.95–1.27)
Q5	1.14 (1.01–1.29)*	1.35 (1.18–1.55)**	1.21 (1.04–1.40)*	1.23 (1.07–1.42)*	1.25 (1.09–1.42)*
*P* for trend	0.194	<0.001	0.042	0.177	0.080
Number of physicians per 10,000 population					
Q1	1.00	1.00	1.00	1.00	1.00
Q2	1.19 (1.08–1.31)*	1.11 (0.98–1.24)	1.13 (1.02–1.25)*	1.14 (1.04–1.25)*	1.15 (1.05–1.26)*
Q3	1.09 (0.98–1.21)	1.07 (0.98–1.17)	1.09 (0.99–1.21)	1.06 (0.95–1.18)	1.05 (0.94–1.18)
Q4	1.21 (1.12–1.31)**	1.20 (1.08–1.32)**	1.18 (1.05–1.33)*	1.13 (1.05–1.22)*	1.12 (0.99–1.26)
Q5	1.25 (1.16–1.35)**	1.25 (1.15–1.35)**	1.24 (1.12–1.38)**	1.21 (1.14–1.29)**	1.21 (1.10–1.33)**
*P* for trend	<0.001	<0.001	<0.001	<0.001	<0.001

CI, confidence interval; PR, adjusted prevalence ratio; Q1, 1st quintile group; Q2, 2nd quintile group; Q3, 3rd quintile group; Q4, 4th quintile group; Q5, 5th quintile group. *P < 0.05, **P < 0.001.

Outcome was the logarithm of the cumulative number of fully vaccinated people aged 65 and older as of July 6, 2021 by prefecture, with the logarithm of the prefectural population included as an offset term.

^a^Adjusted for logarithm of population density, influenza vaccination coverage, household income, annual mean temperature, and all-cause mortality.

^b^Adjusted for logarithm of population density, influenza vaccination coverage, household income, annual mean temperature, all-cause mortality, and the number of civil servants and physicians (i.e., mutually adjusted for the number of civil servants and physicians, in addition to the variables in Model 0).

^c^Adjusted for logarithm of population density, influenza vaccination coverage, educational status, annual mean relative humidity, cancer incidence rate, and the number of civil servants and physicians.

^d^Adjusted for logarithm of population density, influenza vaccination coverage, Gini coefficient, annual sunshine hours, per capita medical costs, and the number of civil servants and physicians.

In an additional analysis using the number of other medical resources as a substitute for the number of physicians, a significant difference between the 1st quintile group and the 5th quintile group was found in the number of clinics and the number of hospitals in Model 1, but the number of nurses had no significant association in all models (Additional file 4). The results for *P* for trend were significant only for the number of clinics in all models, but not for the number of hospitals and the number of nurses in either model. These results showed that the number of clinics was strongly associated with the rapid vaccination of older people.

In an analysis stratifying the number of civil servants into prefectural civil servants and municipal civil servants, a significant difference between the 1st quintile group and the 5th quintile group was observed for prefectural civil servants in Model 3 and for municipal civil servants in Models 1 and 2 (Additional file 5). The results for *P* for trend were significant for municipal civil servants in Models 2 and 3, but not for the number of prefectural civil servants in either model. These results suggested that the number of municipal civil servants was strongly associated with the rapid vaccination of older people.

## Discussion

This study investigated the cross-sectional relationship of the number of civil servants and physicians with the prefectural-level rapid COVID-19 vaccination of older people based on an ecological analysis. COVID-19 vaccination was found to be more rapid in prefectures with a large number of civil servants and physicians. A significant trend between higher staffing levels and more rapid vaccination was seen in all models for the number of physicians, but only in Model 1 for the number of civil servants. As far as we know, this study was the first to suggest that the number of civil servants and physicians per population was associated with rapid COVID-19 vaccine uptake among older people, and that the number of physicians had a stronger association.

The mechanism of the association of the number of civil servants and physicians with rapid COVID-19 vaccination is not fully understood, but we suggest the following as possible explanations. In Japan, local government officials are responsible for managing the COVID-19 vaccination program (sending vaccination tickets, establishing a vaccination booking system, arranging medical staff, and setting up vaccination centers, etc.) [6, 9, 12]. Therefore, COVID-19 vaccination may have progressed rapidly in prefectures with a large number of civil servants who are in charge of the vaccination program. Moreover, COVID-19 vaccination may have proceeded quickly in prefectures with a large number of physicians who administer vaccines. A previous study in Japan reported that the presence of a family physician was associated with routine vaccination in older people, suggesting that family physicians play a key role in expediting COVID-19 vaccination among older people [7]. In Japan, in order to expedite vaccination of the people, mass vaccination centers have been set up to make vaccinations more accessible [9]. However, since many older people have difficulty getting to a vaccination center, they are often vaccinated by a practitioner near their home, and as a result, family physicians may become indispensable providers of vaccinations for older people. This explanation is also supported by our additional analyses showing that the number of clinics was more strongly associated with rapid vaccination in older people than the number of hospitals.

Our study has strengths. First, the open data used in this survey is based on statistical surveys conducted by Japanese government agencies and, as prefectural-level data, has high representativeness. Second, our study succeeded in adjusting for population density, influenza vaccination coverage, socioeconomic factors, natural environmental factors, and health indicators, which are important confounders of the relationship between the number of civil servants and physicians and vaccination status [21, 29, 30].

Our study has limitations. First, since this study is an ecological study, there are limitations such as the possibility of ecological fallacy, the inability to adjust for the necessary confounding factors, and the inability to identify causal relationships. Second, vaccinations based on the Preventive Vaccination Act are conducted on a municipal basis, which is the responsibility of the mayor of the municipality. Ideally, analysis should be conducted on a municipal basis. However, because the daily number of vaccinated persons was not published by municipality, but available by prefecture, this study used data at the prefectural level. Several prefectures [31, 32] have published their latest COVID-19 vaccination coverage by municipality, and this data shows that there are differences in vaccination status among municipalities within the same prefecture. For example, it is conceivable that differing views among mayors of municipalities contributed to the vaccine policy. Therefore, it should be noted that the results of this study do not take into account differences between municipalities. Third, although we used as many covariates as possible, this study has the potential for unmeasured confounding. For example, previous studies reported that knowledge about vaccine efficacy and trust in the authorities were important factors for COVID-19 vaccine intent [8, 29, 33], but this study has failed to adjust for these factors. Fourth, in the recent vaccination program in prefectures, the personnel system has been supported by departments other than the department in charge of vaccination in normal times, but non-regular workers such as dispatched workers have also played a major role [6, 34]. In addition, municipalities actively outsourced work that could be outsourced, and secured the necessary personnel to implement vaccinations [6]. Therefore, it should be noted that the number of civil servants used in this study does not reflect the total number of personnel involved, such as dispatched workers or outsourced workers.

In conclusion, our study showed that prefectures with a large number of civil servants and physicians per population had rapid COVID-19 vaccine uptake among older people. Our results suggest that civil servants and physicians may be key human resources in the rapid rollout of COVID-19 vaccinations among older people in Japan. This study may point the way to future areas of research on vaccine policies that include other age groups and infectious diseases.

## Abbreviations

CI: confidence interval
COVID-19: Coronavirus disease 2019
PR: prevalence ratio
SD: standard deviation
VRS: Vaccination Record System

## Supplementary information

**Additional file 1** Definition and data source of covariates. **Additional file 2** Reasons for adoption as a covariate. **Additional file 3** Data of outcome and explanatory variables in 47 prefectures. **Additional file 4** Association of the number of medical resources per 100,000 population with rapid COVID-19 vaccination in older people: Additional analyses used the number of other medical resource as a substitute for the number of physicians. **Additional file 5** Association of the number of civil servants per 1,000 population with rapid COVID-19 vaccination in older people: Stratified analyses by prefectures and municipalities.

## Declarations

### Availability of data and materials

All data used in this study are publicly available. Data can be downloaded from the following websites: vaccination status (https://info.vrs.digital.go.jp/dashboard/), the number of civil servants (https://www.e-stat.go.jp/stat-search/files?page=1&layout=datalist&toukei=00200212&tstat=000001164275&cycle=0&tclass1=000001164517&tclass2val=0), and the number of physicians (https://www.mhlw.go.jp/toukei/saikin/hw/ishi/20/index.html). Details of the data source of each variable adopted as a covariate in this study are presented in Additional file 1.
