# Supplementary material for: An ecological association of number of civil servants and physicians with prefectural-level rapid COVID-19 vaccination of older people in Japan
Source: Environ Health Prev Med. 2023 Apr 7;28:21. doi: 10.1265/ehpm.22-00145 (PMC10106328; doi:10.1265/ehpm.22-00145)
Supplement: Supplementary file 1 — Additional file 1 Definition and data source of covariates. Additional file 2 Reasons for adoption as a covariate. Additional file 3 Data of outcome and explanatory variables in 47 prefectures. Additional file 4 Association of the number of medical resources per 100,000 population with rapid COVID-19 vaccination in older people: Additional analyses used the number of other medical resource as a substitute for the number of physicians. Additional file 5 Association of the number of civil servants per 1,000 population with rapid COVID-19 vaccination in older people: Stratified analyses by prefectures and municipalities. [file ehpm-28-021-s001.pdf]

Additional file 1. Definition and data source of covariates

Category	Variable	Definition	Data source	Implementing body	Year
Common to all models	Population density	Logarithm of population density (population per square kilometer)	National Population Census	Ministry of Internal Affairs and Communications	2020
	Influenza vaccination coverage	Proportion of the number of older people who received routine influenza vaccinations administered by municipalities divided by the population of older people aged 65 and older	Community Health and Health Promotion Project Report	Ministry of Health, Labour and Welfare	2019
Socioeconomic factors	Household income	Equivalized household disposable income adjusted by regional price parities (100,000 yen per year)	National Survey of Family Income, Consumption and Wealth	Ministry of Internal Affairs and Communications	2019
	Educational status	Proportion of upper secondary graduates going to further education	School Basic Survey	Ministry of Education, Culture, Sports, Science and Technology	2021
	Gini coefficient	Gini coefficient of equivalized yearly disposable income	National Survey of Family Income, Consumption and Wealth	Ministry of Internal Affairs and Communications	2019
Natural environmental factors	Annual mean temperature	Yearly average of air temperature	Past Weather Data	Japan Meteorological Agency	2020
	Annual mean relative humidity	Yearly average of relative humidity	Past Weather Data	Japan Meteorological Agency	2020
	Annual sunshine hours	Total number of yearly sunshine hours (100 hours)	Past Weather Data	Japan Meteorological Agency	2020
Health indicators	All-cause mortality	Age-adjusted mortality from all causes, inclusive of men and women (base period population = 1930) (per 1,000 persons)	Vital Statistics Special Report	Ministry of Health, Labour and Welfare	2015
	Cancer incidence rate	Age-adjusted cancer incidence rate in both genders (standard population = 1985 Japanese model population) (per 1 million)	Cancer Statistics (National Cancer Registry)	National Cancer Center Japan	2019
	Per-capita medical costs	Average annual medical expenses per person (10,000 yen)	National medical expenses	Ministry of Health, Labour and Welfare	2019

Additional file 2. Reasons for adoption as a covariate

For socioeconomic factors, we adopted household income, educational status, and Gini coefficient, because they were significant confounding factors in previous studies examining COVID-19 vaccine acceptance and preferences in Japan [1, 2], and important explanatory variables in an ecological study reporting socioeconomic disparities in the COVID-19 incidence rate in Japan [3]. For educational status, a previous ecological study[3] used percentage of graduates aged 20 years or older with a college or a higher-level degree based on the Employment Status Survey. The Employment Status Survey is conducted by the Ministry of Internal Affairs and Communications every five years, and the latest published data is 2017, which is old. On the other hand, the School Basic Survey is conducted every year by the Ministry of Education, Culture, Sports, Science and Technology, and the data on the education continuance rate by prefecture for 2021 is available. Therefore, in this study, we used the proportion of upper secondary graduates going to further education by prefecture based on the Basic School Survey.

For natural environmental factors, we adopted annual mean temperature, annual mean relative humidity, and annual sunshine hours. Previous studies reported that temperature and humidity were associated with the spread and severity of COVID-19 at the local level [4, 5]. Therefore, we used the annual mean temperature and annual mean

relative humidity published by the Japan Meteorological Agency. Furthermore, although UV radiation has been reported to be an important factor in the transmission of COVID-19 [6], there is no open data on UV radiation per prefecture in Japan. Because the intensity of UV rays is determined by the amount of ozone in the stratosphere and the duration of sunshine [7], this study used the annual sunshine hours per prefecture published by the Japan Meteorological Agency.

For health indicators, we adopted all-cause mortality, cancer incidence rate, and per capita medical costs. Previous studies have reported that health conditions are associated with acceptance and hesitancy of COVID-19 vaccine [1, 2]. For example, people with underlying medical conditions or poor subjective health status are willing to receive the COVID-19 vaccine. In this study, we used all-cause mortality, cancer incidence rate, and per capita medical costs as indices representing health conditions at the prefecture level.

References

1. Kadoya Y, Watanapongvanich S, Yuktadatta P, Putthinun P, Lartey ST, Khan MSR. Willing or Hesitant? A Socioeconomic Study on the Potential Acceptance of COVID-19 Vaccine in Japan. Int J Environ Res Public Health. 2021;18:4864.

2. Kawata K, Nakabayashi M. Determinants of COVID-19 vaccine preference: A survey study in Japan. SSM Popul Health. 2021;15:100902.
3. Yoshikawa Y, Kawachi I. Association of Socioeconomic Characteristics With Disparities in COVID-19 Outcomes in Japan. JAMA Netw Open. 2021;4:e2117060.
4. Landier J, Paireau J, Rebaudet S, et al. Cold and dry winter conditions are associated with greater SARS-CoV-2 transmission at regional level in western countries during the first epidemic wave. Sci Rep. 2021;11:12756.
5. Yamasaki L, Murayama H, Hashizume M. The impact of temperature on the transmissibility potential and virulence of COVID-19 in Tokyo, Japan. Sci Rep. 2021;11:24477.
6. Scabbia G, Sanfilippo A, Mazzoni A, et al. Does climate help modeling COVID-19 risk and to what extent? PLoS One. 2022;17:e0273078.
7. Japan Meteorological Agency. Data on UV rays. https://www.data.jma.go.jp/gmd/env/uvhp/info_uv.html. Accessed 25 Oct 2022. (Japanese).

Additional file 3. Data of outcome and explanatory variables in 47 prefectures.

Prefectures		Outcome: No. of fully vaccinated older people, as of July 6, 2021	Outcome: Population aged ≥65 in 2021 (unit: 1,000 people)	Explanatory: No. of people per population: Civil servants per 1,000 population	Explanatory: No. of people per population: Physicians per 10,000 population
1	Hokkaido	623,914	1,686	27.0	25.1
2	Aomori	177,362	419	30.2	21.3
3	Iwate	155,390	409	31.6	20.7
4	Miyagi	332,105	655	24.2	24.6
5	Akita	111,227	360	28.5	24.3
6	Yamagata	187,565	362	30.3	22.9
7	Fukushima	272,079	585	26.1	20.6
8	Ibaraki	322,047	860	21.0	19.4
9	Tochigi	191,462	569	20.6	23.7
10	Gumma	233,130	589	23.2	23.4
11	Saitama	776,874	2,000	16.8	17.8
12	Chiba	671,796	1,748	18.6	20.6
13	Tokyo	1,495,342	3,202	19.1	32.1
14	Kanagawa	818,379	2,376	16.7	22.3
15	Niigata	337,213	723	26.0	20.4
16	Toyama	149,702	337	27.2	26.2
17	Ishikawa	178,952	338	26.0	29.2
18	Fukui	114,966	236	29.7	25.8
19	Yamanashi	106,755	252	29.5	25.0
20	Nagano	305,059	657	26.0	24.4
21	Gifu	315,611	605	23.8	22.5
22	Shizuoka	387,714	1,099	21.1	21.9
23	Aichi	913,660	1,918	20.0	22.4
24	Mie	263,509	531	24.4	23.2
25	Shiga	166,812	376	24.1	23.6
26	Kyoto	366,073	758	21.6	33.3
27	Osaka	1,104,937	2,442	18.3	28.6
28	Hyogo	702,176	1,608	21.4	26.6
29	Nara	183,269	423	23.7	27.7
30	Wakayama	177,545	308	30.8	30.8
31	Tottori	89,135	180	34.3	31.5
32	Shimane	101,585	229	35.3	29.7
33	Okayama	301,187	575	22.6	32.0
34	Hiroshima	308,706	827	21.1	26.7
35	Yamaguchi	244,692	465	25.2	26.0
36	Tokushima	127,380	247	31.2	33.8
37	Kagawa	146,334	303	27.3	29.0
38	Ehime	188,009	444	26.4	27.7
39	Kochi	128,868	245	36.2	32.2
40	Fukuoka	659,262	1,445	17.8	31.0
41	Saga	142,741	251	27.3	29.0
42	Nagasaki	198,526	435	26.4	31.9
43	Kumamoto	271,935	551	24.5	29.7
44	Oita	178,311	376	25.9	28.7
45	Miyazaki	166,130	351	26.2	25.6
46	Kagoshima	234,152	521	27.6	28.4
47	Okinawa	124,579	339	26.0	25.7

Additional file 4. Association of the number of medical resources per 100,000 population with rapid COVID-19 vaccination in older people: Additional analyses used the number of other medical resources as a substitute for the number of physicians.

	Explanatory: No of clinics per population				Explanatory: No of hospitals per population				Explanatory: No of nurses per population		
	Model 1	Model 2	Model 3		Model 1	Model 2	Model 3		Model 1	Model 2	Model 3
	APR[a] (95% CI)	APR[b] (95% CI)	APR[c] (95% CI)		APR[a] (95% CI)	APR[b] (95% CI)	APR[c] (95% CI)		APR[a] (95% CI)	APR[b] (95% CI)	APR[c] (95% CI)
Number of clinics				Number of hospitals[d]				Number of nurses			
Q1	1.00	1.00	1.00	Q1	1.00	1.00	1.00	Q1	1.00	1.00	1.00
Q2	0.98 (0.88-1.08)	1.01 (0.91-1.12)	1.02 (0.92-1.13)	Q2	1.08 (0.98-1.20)	1.02 (0.92-1.14)	0.98 (0.84-1.13)	Q2	0.91 (0.81-1.02)	0.90 (0.80-1.02)	0.83 (0.74-0.93)*
Q3	1.08 (0.98-1.20)	1.12 (1.00-1.26)*	1.10 (0.97-1.24)	Q3	1.07 (0.92-1.26)	0.98 (0.84-1.15)	0.96 (0.80-1.14)	Q3	0.84 (0.73-0.97)*	0.86 (0.75-0.99)*	0.78 (0.67-0.92)*
Q4	1.06 (0.95-1.18)	1.08 (0.98-1.19)	1.06 (0.93-1.19)	Q4	1.14 (0.99-1.33)	1.07 (0.93-1.24)	1.03 (0.82-1.29)	Q4	1.02 (0.90-1.16)	1.04 (0.92-1.17)	0.89 (0.75-1.07)
Q5	1.12 (1.01-1.24)*	1.10 (0.99-1.22)	1.08 (0.93-1.26)	Q5	1.27 (1.07-1.51)*	1.14 (0.99-1.32)	1.03 (0.76-1.38)	Q5	0.98 (0.81-1.19)	1.03 (0.90-1.18)	0.83 (0.66-1.04)
	P for trend = 0.024	P for trend = 0.003	P for trend = 0.028		P for trend = 0.246	P for trend = 0.285	P for trend = 0.937		P for trend = 0.687	P for trend = 0.237	P for trend = 0.919

APR, adjusted prevalence ratio; CI, confidence interval; Q1, 1st quintile group; Q2, 2nd quintile group; Q3, 3rd quintile group; Q4, 4th quintile group; Q5, 5th quintile group. $^{*}P < 0.05$.

Outcome was the logarithm of the cumulative number of fully vaccinated people aged 65 and older as of July 6, 2021 by prefecture, with the logarithm of the prefectural population included as an offset term.

[a]Adjusted for logarithm of population density, influenza vaccination coverage, household income, annual mean temperature, all-cause mortality, and the number of civil servants.

[b]Adjusted for logarithm of population density, influenza vaccination coverage, educational status, annual mean relative humidity, cancer incidence rate, and the number of civil servants.

[c]Adjusted for logarithm of population density, influenza vaccination coverage, Gini coefficient, annual sunshine hours, per capita medical costs, and the number of civil servants.

[d]Excluding the number of psychiatric hospitals and tuberculosis hospitals.

Additional file 5. Association of the number of civil servants per 1,000 population with rapid COVID-19 vaccination in older people: Stratified analyses by prefectures and municipalities

Explanatory: No of prefectural civil servants				Explanatory: No of municipal civil servants			
	Model 1<br>APR[a] (95% CI)	Model 2<br>APR[b] (95% CI)	Model 3<br>APR[c] (95% CI)		Model 1<br>APR[a] (95% CI)	Model 2<br>APR[b] (95% CI)	Model 3<br>APR[c] (95% CI)
Number of civil servants in prefectures				Number of civil servants in municipalities			
Q1	1.00	1.00	1.00	Q1	1.00	1.00	1.00
Q2	1.03 (0.95-1.11)	1.05 (0.98-1.13)	1.07 (0.99-1.15)	Q2	1.10 (1.02-1.19)*	1.12 (1.02-1.22)*	1.11 (1.01-1.22)*
Q3	1.12 (1.02-1.22)*	1.17 (1.07-1.29)*	1.18 (1.08-1.29)**	Q3	1.14 (1.03-1.27)*	1.16 (1.04-1.29)*	1.16 (1.04-1.30)*
Q4	1.05 (0.95-1.15)	1.07 (0.97-1.19)	1.06 (0.96-1.18)	Q4	1.05 (0.96-1.15)	1.07 (0.98-1.17)	1.07 (0.98-1.17)
Q5	1.07 (0.96-1.19)	1.11 (0.997-1.24)	1.14 (1.02-1.27)*	Q5	1.13 (1.01-1.27)*	1.14 (1.01-1.29)*	1.13 (0.995-1.28)
	P for trend = 0.084	*P* for trend = 0.182	*P* for trend = 0.085		*P* for trend = 0.096	*P* for trend = 0.049	*P* for trend = 0.039

APR, adjusted prevalence ratio; CI, confidence interval; Q1, 1st quintile group; Q2, 2nd quintile group; Q3, 3rd quintile group; Q4, 4th quintile group; Q5, 5th quintile group. *$P<0.05$, **$P<0.001$.

Outcome was the logarithm of the cumulative number of fully vaccinated people aged 65 and older as of July 6, 2021 by prefecture, with the logarithm of the prefectural population included as an offset term.

Municipalities include ordinance-designated cities and special wards of Tokyo.

[a]Adjusted for logarithm of population density, influenza vaccination coverage, household income, annual mean temperature, all-cause mortality, and the number of civil servants.

[b]Adjusted for logarithm of population density, influenza vaccination coverage, educational status, annual mean relative humidity, cancer incidence rate, and the number of civil servants.

[c]Adjusted for logarithm of population density, influenza vaccination coverage, Gini coefficient, annual sunshine hours, per capita medical costs, and the number of civil servants.
